# Sensory Drive Mediated by Climatic Gradients Partially Explains Divergence in Acoustic Signals in Two Horseshoe Bat Species, *Rhinolophus swinnyi* and *Rhinolophus simulator*

**DOI:** 10.1371/journal.pone.0148053

**Published:** 2016-01-27

**Authors:** Gregory L. Mutumi, David S. Jacobs, Henning Winker

**Affiliations:** 1 Animal Evolution and Systematics Group (AES), Biological Sciences Department, University of Cape Town, Cape Town 7701, South Africa; 2 Centre for Statistics in Ecology, Environment and Conservation (SEEC), South African National Biodiversity Institute (SANBI), Cape Town, South Africa; University of Arkansas, UNITED STATES

## Abstract

Geographic variation can be an indicator of still poorly understood evolutionary processes such as adaptation and drift. Sensory systems used in communication play a key role in mate choice and species recognition. Habitat-mediated (i.e. adaptive) differences in communication signals may therefore lead to diversification. We investigated geographic variation in echolocation calls of African horseshoe bats, *Rhinolophus simulator* and *R*. *swinnyi* in the context of two adaptive hypotheses: 1) James’ Rule and 2) the Sensory Drive Hypothesis. According to James’ Rule body-size should vary in response to relative humidity and temperature so that divergence in call frequency may therefore be the result of climate-mediated variation in body size because of the correlation between body size and call frequency. The Sensory Drive Hypothesis proposes that call frequency is a response to climate-induced differences in atmospheric attenuation and predicts that increases in atmospheric attenuation selects for calls of lower frequency. We measured the morphology and resting call frequency (RF) of 111 *R*. *simulator* and 126 *R*. *swinnyi* individuals across their distributional range to test the above hypotheses. Contrary to the prediction of James’ Rule, divergence in body size could not explain the variation in RF. Instead, acoustic divergence in RF was best predicted by latitude, geography and climate-induced differences in atmospheric attenuation, as predicted by the Sensory Drive Hypothesis. Although variation in RF was strongly influenced by temperature and humidity, other climatic variables (associated with latitude and altitude) as well as drift (as suggested by a positive correlation between call variation and geographic distance, especially in *R*. *simulator*) may also play an important role.

## Introduction

Variation in phenotypic characteristics across the distributional range of a species is common to all organisms. Such geographic variation in phenotype could involve morphological features as well as behavioural characteristics such as sensory modalities (e.g., echolocation), foraging habitat and prey preferences. Variation in such characters over the distributional range of a species can be the result of dispersal and adaptation to novel environments [1,2,3]. However, it may also be the result of stochastic factors such as random genetic drift especially when populations are small such as during founder events when new populations are established [4].

Although drift and selection can both play roles in the evolutionary history of organisms, traits that are heritable and have strong impacts on fitness, are less likely to be impacted by drift, unless populations are small [4,5]. Thus geographic variation in traits associated with sensory systems employed in communication is likely to be adaptive because they play a key role in mate choice [6] and species recognition [7]. Communication signals (be they visual, olfactory or acoustic) have to be produced, transmitted and perceived under prevailing local environmental conditions while remaining relevant and easy to detect. The importance of the role that the environment, particularly climate, plays in such acoustic signal variation is increasingly being recognized [8,9]. This has resulted in the formulation of the Sensory Drive Hypothesis which proposes that lineage diversification may be driven by environmentally-mediated differences in communication signals [10]. This hypothesis thus predicts an adaptive, rather than stochastic, response in acoustic signals to environmental variables.

Complex signals with high information content, such as bird song and the echolocation calls of bats, are particularly sensitive to environmental conditions [11]. Echolocation calls are used for prey detection and orientation [12,13] which have an ecological context making echolocation ideal for the study of geographic variation influenced by environmental factors. Furthermore, evidence is emerging that bats also use echolocation for communication [12,14,15,16] and echolocation may play a role in mate choice. If so, echolocation may be implicated in lineage diversification [6].

Acoustic signals may diverge along climatic gradients as a result of variation in atmospheric attenuation of sound. Atmospheric attenuation, the decrease in the energy of a sound as a result of scattering and absorption by the atmosphere, is the result of a complex interaction between humidity, temperature and the frequency of the sound [8,17]. For example, wood warblers and bats of the American south-west used lower frequencies in more humid environments (absorption is high) to optimise sound propagation [18]. In bats, differences in humidity and temperature across the geographic range of a species may select for different echolocation frequencies so that atmospheric attenuation due to these climatic factors is minimized and the detection range of echolocation is optimized. Furthermore, because higher frequency sound is attenuated to a greater degree than lower frequency sound [17], variation in the frequency of echolocation as a result of attenuation is likely to be more pronounced in bat species using calls of high frequency. Previous field studies e.g. Guillén *et al*. and Jiang *et al*. [19,20] have focused on how the frequencies of acoustic signals change in response to changes in humidity but have ignored the effects of call frequency and temperature on atmospheric attenuation. Here we considered all three components of atmospheric attenuation under the Sensory Drive Hypothesis by comparing the effects of temperature and humidity on the call frequencies of two species of bats with very different mean echolocation frequencies. The adaptive response predicted by the Sensory Drive Hypothesis should result in lower call frequencies in habitats with higher atmospheric attenuation (lower temperature and higher humidity) and this effect should be more pronounced for calls of higher frequency.

There has been evidence for an inverse relationship between body size and echolocation frequency [21,22] as well as between body size and humidity and this may confound the relationship between echolocation frequency and climatic factors. HHhhhJames’ Rule [23], proposes that animals in hot humid environments generally have smaller body sizes than animals of the same species that occur in cooler, humid areas, and the largest animals are expected to occur in cool, dry areas. This would in turn lead to differences in other morphological parameters if allometry is maintained. James’ Rule thus predicts smaller body sizes in hotter, more humid environments which should result in higher call frequencies. This is opposite to the relationship predicted for these two variables by the Sensory Drive Hypothesis.

The objectives of our study were to test the validity of sensory drive as an explanation for divergence in acoustic signals using two horseshoe bat species of similar size but with different echolocation frequencies. This minimized the effects of size on echolocation variation, allowing us to test the influence of atmospheric attenuation on calls of different frequencies. We assessed 1) the level of geographic variation present in echolocation frequency in each species and 2) the contributions of environmental variables and body size to call frequency divergence as predicted under James’s Rule and the Sensory Drive Hypotheses.

## Methods

### Study animals

**Fig 1:**
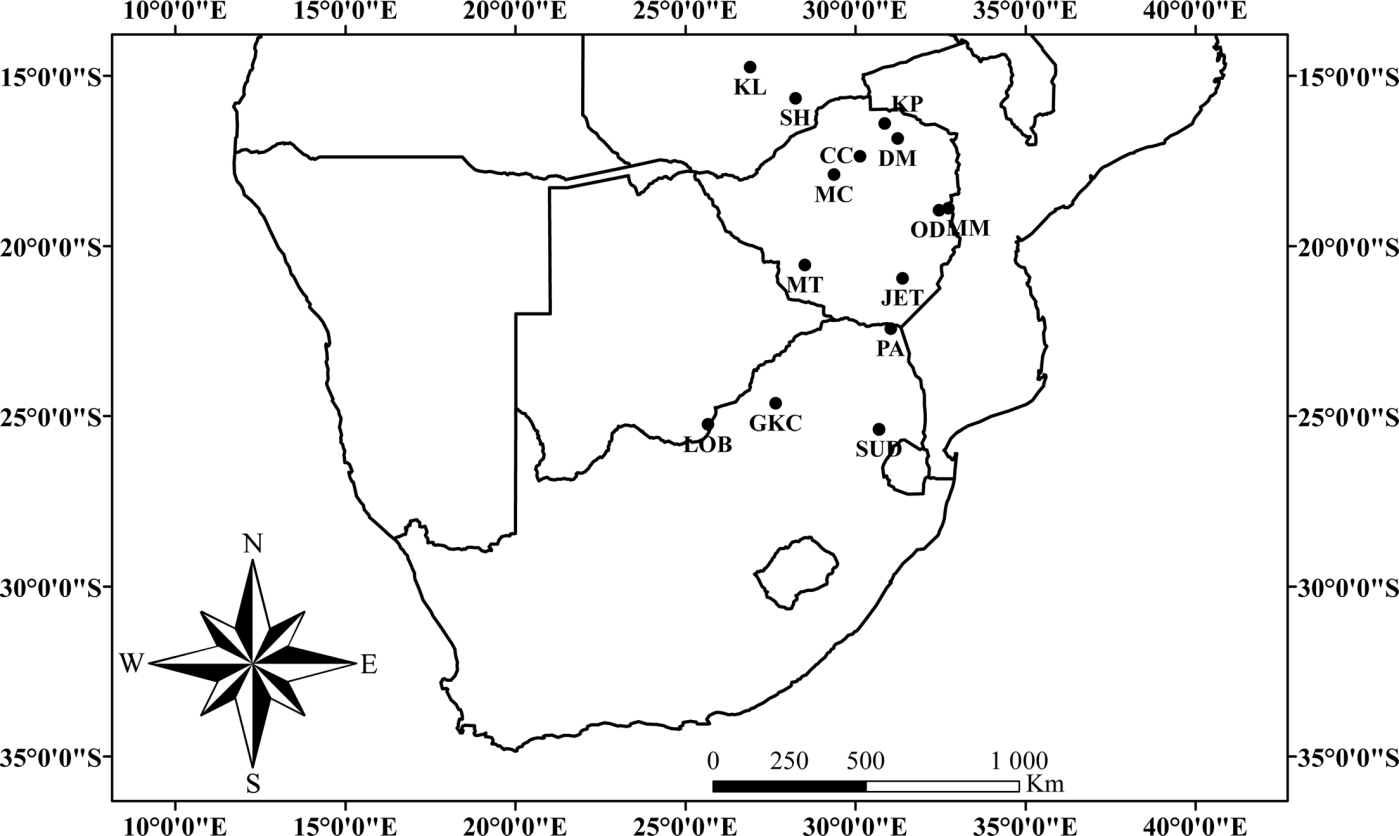
Sampling sites within southern Africa from where *R*. *simulator* and *R*. *swinnyi* were caught. Abbreviations: CC = Chinhoyi, Zimbabwe; DM = Dambanzara, Zimbabwe; GKC = Gatkop Cave, South Africa; JET = Jiri Estate Triangle, Zimbabwe; KL = Kalenda, Zambia; KP = Kapatamukombe, Zimbabwe; LOB = Lobatse Estate, Botswana; MC = Mabura Cave, Zimbabwe; MM = Monaci Mine, Zimbabwe; MT = Matobo Hills, Zimbabwe; OD = Odzi German Shafts, Zimbabwe; PA = Pafuri, South Africa; SH = Shimabala, Zambia; and SUD = Sudwala, South Africa.

We focused on two species of insectivorous horseshoe bats *Rhinolophus simulator* and *R*. *swinnyi* which use high duty cycle (signal duration is long compared to the silent period) echolocation calls dominated by a constant frequency component (S1 Fig) at means of 80 kHz and 107 kHz, respectively [24]. Both species inhabit heterogeneous habitats within a savannah biome and are widely distributed throughout the more mesic eastern half of southern and central Africa [25]; Fig 1. The savannah biome is composed of several woodland types with unique vegetation and climate, commonly classified into ecoregions [26]. *Rhinolophus simulator* has a similar distribution to that of *R*. *swinnyi* but extends further north into central Ethiopia through western Kenya and central Tanzania [25].

### Ethical Statement

Capture, handling and voucher collection methods of this research complied with the guidelines recommended by the American Society of Mammalogists [27], and sampling guidelines compiled by Aegerter *et al*. [28] and Kunz & Parsons [29], and were approved by the Science Faculty Animal Ethics Committee at the University of Cape Town (Clearance Number 2013/2011/V6/DJ). All workers handling bats were vaccinated for rabies and were required to use protective gloves when handling bats and samples. All sampling was on non-protected species (S1 Table) captured from both privately owned and protected areas under the authority of: Zimbabwe (Parks and Wildlife Management Authority + Permit [23 (1) (C) (II) 25/2011; 19/2012 and 16/2013], South Africa (Northern Cape Province, Fauna 764/2010; Mupumalanga Tourism & Park Agency, MPB 5253; Cape Nature, 0035-AAA007-00081), Malawi (Department of Forestry Licence NO: 1/06/2013/1), Botswana (Ministry of Environment, Wildlife and Tourism, EWT 8/36/4 XVI – 78).

### Sampling

Bats were caught from caves and disused mine-shafts at 14 locations (Fig 1) across the distributional ranges of the focal species along a latitudinal gradient ranging from 16°S to 32°S (Fig 1). Hand-nets and continuously monitored harp-traps and mist-nets were used at cave and mine exits and where possible, within caves and mines. After capture, bats were held individually in soft cotton bags. Sex and reproductive status were checked immediately following capture and bats in late pregnancy or early lactation were released. Reproductive status was determined by examination of the nipples and palpation of the abdomen of female bats [30]. Juveniles were distinguished from adults by the presence of cartilaginous epiphyseal plates in their finger bones detected by trans-illuminating the bat’s wings [31]. Only non-pregnant/lactating adults were used in subsequent analyses.

Forearm length (FA) was measured to the nearest 0.1 mm using dial callipers and body mass (to the nearest 0.5 g) using a portable electronic balance. We chose FA as a measure of body size instead of mass because FA is not prone to seasonal and diurnal fluctuations [32].

Echolocation calls were recorded (for approximately 45 seconds per bat) from hand-held individuals 30 cm from an Avisoft UltraSoundGate 416 (Avisoft Bioacoustics, Berlin, Germany), using a condenser ultrasound microphone (Avisoft-Bioacoustics CM16/CMPA). Calls were recorded onto an HP Compaq nx7010 notebook computer with RECORDER USGH Software from Avisoft. Hand-held calls allow the determination of the resting peak frequency (RF; frequency of maximal energy when at rest) in rhinolophid bats [33] and eliminate variation in peak frequency as a result of horseshoe bats compensating for Doppler shifts during flight [34]. Recordings were slowed down by 10x and analysed using BatSound Pro software (version 3.20, Pettersson Elektronik AB, Uppsala, Sweden) using a sampling frequency of 500 kHz, a resolution of 16 bits mono and a threshold of 15. The frequency of the dominant 2^nd^ harmonic of high-quality calls (i.e., high signal-to-noise ratio) were measured from the power spectrum using a Hanning window and the duration of calls were measured from the oscilloscope. The first 10 calls in each recorded sequence were not analysed because horseshoe bats tune into their RF after periods of silence [33]. The constant frequency component of the calls usually stabilizes (i.e., little to no variation in the frequency) by the 11^th^ call. Ten calls were selected for analyses and an average RF calculated for each bat. The frequency of an actual call closest to the average RF of these ten calls was used in subsequent analyses for each bat.

### Environmental Variables

ArcGIS Shape files were downloaded from BIOCLIM (**http://www.worldclim.org/bioclim**) and OEI (www.en.openei.org) websites and analysed in ArcGIS v.10 for the following environmental variables: relative humidity (RH), mean annual temperature (AnnTemp), altitude (Alt), latitude (Lat) and longitude (Long). We used Alt as a proxy for air pressure because a significant relationship between atmospheric pressure, Alt and RF has previously been reported [35,36] and we were unable to obtain air pressure data.

The shape files (at a resolution of 30 arc seconds) for AnnTemp were based on monthly temperature values averaged over 50 years (1950–2000). RH was based on source data taken at 10 m above the surface of the earth by NASA Surface meteorology and Solar Energy (SSE Release 6.0, Data Set; Nov 2007); a shape file based on 22-year monthly and annual average data set (July 1983—June 2005; http://eosweb.larc.nasa.gov/sse/). These data were regional averages, not point data. Coordinates of each study site were taken using a Garmin-GPS unit (model Colorado 300, Garmin International Inc, Kansas).

### Detection Range

To understand how climatic variables have shaped RF in our focal species, we calculated detection distances of ‘prey’ for each population according to the online-calculator method developed in Stilz and Schnitzler [37]. The calculator estimates the detection range using the following variables: 1) atmospheric conditions: RH, AnnTemp, atmospheric pressure in Pascal’s—Pa, 2) sound properties: RF in hertz–Hz and call intensity in decibels dB [SPL root-mean-square (rms)], 3) energy absorption constant of the target–C1, and 4) two-way geometric spreading constant–C2 between a bat and target. It also gives the degree of attenuation in decibels dB [SPL root-mean-square (rms)] over the estimated detection range calculated from the same input as above.

For prey (the target), the function point-reflector, which best explains the differences in detection ranges for insects was used [37]. The maximum range at which a bat detects an echo from a target depends on the size of the target; the smaller the target, the weaker the echo and is dependent on the specific frequency used by the bat. We used our measured RFs for the sound frequency input. Call intensity of the similar sized horseshoe bat *R*. *blassi*, 117 dB (SPL rms); calculated at a distance of 10 cm from the bats’ nose [38] was used because the intensity of echolocation calls of *R*. *simulator* and *R*. *swinnyi* are currently unknown. Atmospheric pressure was kept at the normal atmospheric condition of 1.013 x 10^5^ Pa [36,39]. The online software has an inbuilt algorithm to calculate target strength (C1) and geometric spreading loss (C2) depending on one’s choice of the target reflecting the pulse. Accordingly, the average detection ranges of our two species at different localities were calculated.

### Statistical analyses

**Fig 2:**
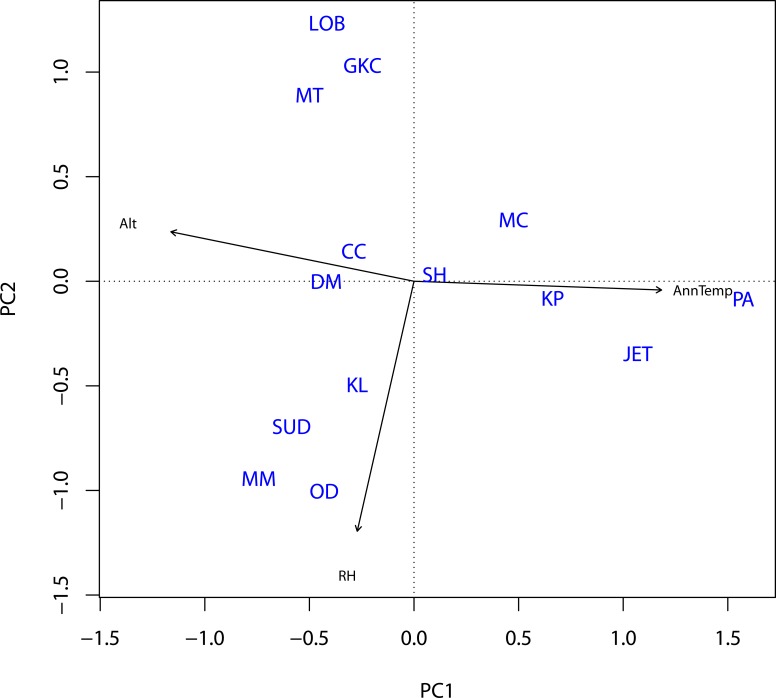
Variation in environmental conditions (relative humidity; RH, mean annual temperature; AnnTemp and altitude; Alt) across sites from which *Rhinolophus simulator* and *R*. *swinnyi* were captured (based on principle component analysis). AnnTemp-PC1 and RH-PC2 accounted for 96% of the variation. Site abbreviations are the same as in Fig 1.

To account for potential multi-collinearity among RH, AnnTemp and Alt [8], and the interactive effect that these variables have on atmospheric attenuation, we used principal component analysis (PCA) to generate uncorrelated variables in the form of principal component scores; PCs [40]. The PCA results suggested that RH, AnnTemp and Alt across study sites could best be summarized as AnnTemp-PC1 and RH-PC2 based on their dominant eigenvalues (Fig 2), which combined accounted for 96% (AnnTemp-PC1 = 63% and RH-PC2 = 33%) of the variation. We preferred to include Alt in the PCA and to keep Lat and Long as separate predictor variables because Alt was likely to carry a strong climate component associated with less spatial dependency (the higher you go, the cooler it becomes) than the actual spatial coordinates. For subsequent analysis, we therefore assumed that the PCs derived from RH, AnnTemp and Alt represent a potential latent effect of environmental variation, whereas Lat and Long were included as spatial predictor variables to account for possible larger scale spatial structuring of our sampling sites as a function of distance.

To construct models for testing the Sensory Drive Hypothesis, we used linear mixed effects models (LMEs) to relate the response of RF to environmental (AnnTemp-PC1 and RH-PC2) and spatial (Lat and Long) predictor variables, while accounting for the effects FA and Sex.

Initial inspection of residual distribution and quantile-quantile plots revealed that the residuals closely approximated a normal distribution. However, further evaluation of residuals versus fitted values and of correlograms [41] provided strong evidence for spatial structuring of residuals which violates the assumption of independence in the data. To determine the most adequate error structure, we tested models based on the above covariates with and without sampling site as a random effect and in association with either none or one of the following three spatial autocorrelation functions: exponential, spherical and gausian (where distances were specified by Lat and Long). The random effect for sampling site was considered to account for the nested sampling design as a result of sampling several individuals from a single location. Spatial autocorrelation is a common phenomenon in animal ecology [42] given that populations in close proximity to each other are likely to be more similar than those far apart as stated by “the first law of geography” [43]. This effectively decreases the number of degrees of freedom, which in turn increases the likelihood of Type I error (incorrect rejection of the null hypothesis) if not accounted for by adequate residual correlation structure within the model [44].

In addition, we employed conventional Mantel tests to provide further insights into the effect of geographic distances among sampling sites on RF. To do so, the Euclidean distances were calculated from geographic coordinates and RF differences were calculated to represent absolute differences in RF between paired sites. We used a simple pairwise Mantel test on the two dissimilarity matrices whereby RFs and geographic distances across sites were regressed to analyse the associations between RF differences and geographic distances in R version 3.1 using package Ade4 [45]. All the tests used 10000 permutations based on Monte-Carlo simulation tests [46].

For both species, the most parsimonious model structure (the one with the lowest Akaike’s Information Criterion; AICc–adjusted for small sample sizes) was found to be an LME with a random effect for site but without spatial autocorrelation. Inspection of correlograms confirmed that the inclusion of the random-effect sufficiently removed the spatial structuring of residuals (S3 Fig) and standard model validation graphs for residuals showed satisfactory residual dispersal against predicted values and normality (S2 Fig)

To determine the optimal combination of covariates, a forward-backward stepwise model selection (on the global model, i.e., with AnnTemp-PC1, RH-PC2, FA, Sex, Lat and Long as predictors) based on AICc was performed using the stepAIC function of the package MASS adjusted to cater for small sample sizes [47] in R. Based on the retained covariates, the ‘best’ model was summarised statistically with an analysis of variance [48] to determine which variables contributed significantly to the variation in the RF response. Only variables that explained a significant proportion of the variation in RF (*p* < 0.05) were included in the final models. This tested the predictions of the Sensory Drive Hypothesis against alternatives, i.e., whether environmental/climatic factors (relative humidity, mean annual temperature and altitude) or body size (James’ Rule), or other factors (sex, and spatial structuring) best explained the variation in resting frequency across sites.

To illustrate the nature of the relationship between covariates and RF divergence across populations, individual effects were predicted by fixing all covariates other than the effect of interest to standardized values (i.e. means across observations and female for sex). Uncoupling the individual environmental effects AnnTemp, RH and Alt required a two-step approach. First, we generated sets of “standardized” PCs for each variable RH, AnnTemp and Alt by fixing two of the variables to their respective means whilst allowing the other to vary [e.g. the designation PC (Alt) meant that AnnTemp and RH were fixed whilst Alt was varied]. Then, we aligned the ‘standardized’ PCs with the other fixed covariates to predict the environmental effect of interest based on the ‘best’ LME.

## Results

### Geographic variation in resting frequency

We analysed the RF of 111 *R*. *simulator* and 126 *R*. *swinnyi* across 10 and 8 sites, respectively (S1 Table 1a and b). *R*. *simulator* had an average RF of 80.32 ± 2.20 kHz and an average duration of 22.92 ± 9.39 ms. *R*. *swinnyi* had an average RF of 103.77 ± 1.70 kHz and an average duration of 28.07 ± 12.45 ms (S1 Table 1a and b). Mean RF for *R*. *simulator* had a range across populations of approximately 7 kHz and that for *R*. *swinnyi* was 4 kHz.

### Geographic variation in detection range

The inferred average detection range of echolocation signal across populations was longer for *R*. *simulator* (7.86 ± 0.30 m) than for *R*. *swinnyi* (6.13 ± 0.25 m). The range of these values was somewhat similar in magnitude (1 m) across populations from 7.5–8.5 m and 5.7–6.5 m, respectively. *R*. *simulator* experienced lower attenuation (average 2.61 dB and range 2.33–2.79 dB across sites) than *Rhinolophus swinnyi* (average 3.68 dB and range 3.37–4.05 dB across sites). Thus there was an increase of ~ 1.01 dB in attenuation across a difference of 27 kHz (80 kHz and 107 kHz for *R*. *simulator* and *R*. *swinnyi*, respectively) between the echolocation frequencies of the two species.

### Effects of environmental variables on RF

For both species, environmental variation (RH, AnnTemp and Alt; comprising AnnTemp-PC1 and RH-PC2) as well as latitude and gender explained the variation in RF. In contrast, body size (FA) did not. Initial model selection using stepAICc dropped FA for *R*. *simulator* and dropped Long & the interaction ‘AnnTemp-PC1: RH-PC2’ for *R*. *swinnyi* (Table 1). ANOVA on the variables maintained in the ‘best’ model for each species yielded the interaction AnnTemp-PC1: RH-PC2, Lat and Sex as significant predictor variables for *R*. *simulator*; whereas for *R*. *swinnyi*, AnnTemp-PC1, RH-PC2, Lat and Sex were significant predictor variables (Table 1). Only the significant variables were used further for predictive modelling.

**Table 1:** The ‘best’ model from forward-backward stepwise model selection on the global model of environmental variables, body size and sex against resting frequency for each of the two species, *Rhinolophus simulator* and *R*. *swinnyi*. Statistics are only presented for variables maintained in the best model.

	*Rhinolophus simulator*				*Rhinolophus swinnyi*			
	numDF	denDF	ANOVA F-value	*p*-value	numDF	denDF	ANOVA F-value	*p*-value
**AnnTemp-PC1**	1	95	1.21	0.278	1	113	6.1	< 0.05
**RH-PC2**	1	95	1.03	0.317	1	113	7.6	< 0.01
**AnnTemp-PC1:RH-PC2**	1	95	4.51	< 0.05				
**Lat**	1	95	12.48	< 0.001	1	113	9.3	< 0.01
**Long**	1	95	0.07	0.783				
**Sex**	1	95	12.03	< 0.001	1	113	56.3	< 0.001
**FA**					1	113	0.0	0.828
Total N = 111					Total N = 126			
Number of Groups: 10					Number of groups: 8			

Abbreviations: AnnTemp-PC1 & RH-PC2 = Principle component factor 1 & 2 derived from relative humidity, mean annual temperature and altitude; Lat = Latitude; Long = Longitude; FA = Forearm length; RF = Resting frequency in kHz; numDf = numerator degrees of freedom; denDF = denominator degrees of freedom.

**Fig 3:**
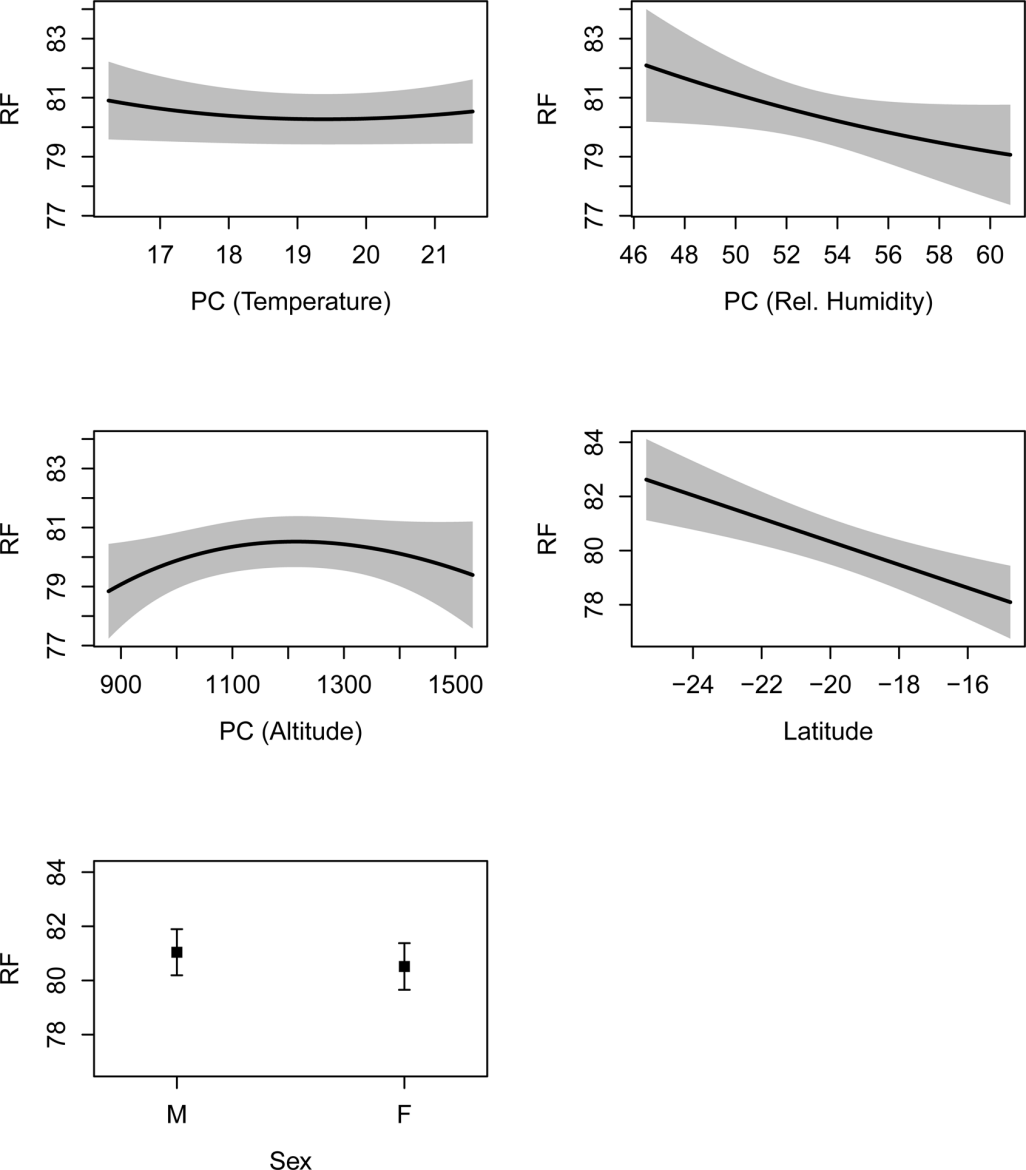
Predictive modelling plots showing how resting frequency (RF) in *Rhinolophus simulator* responded to environmental variation and sex. Abbreviations: e.g., PC (Altitude) represents a principle component generated by fixing mean-annual-temperature and relative humidity (rel.humidity) to their across-site-means while altitude was allowed to vary. The shaded areas and error bars represent 95% confidence limits. The final best model (only variables significant after ANOVA on the best model shown) was used for the modelling.

**Fig 4:**
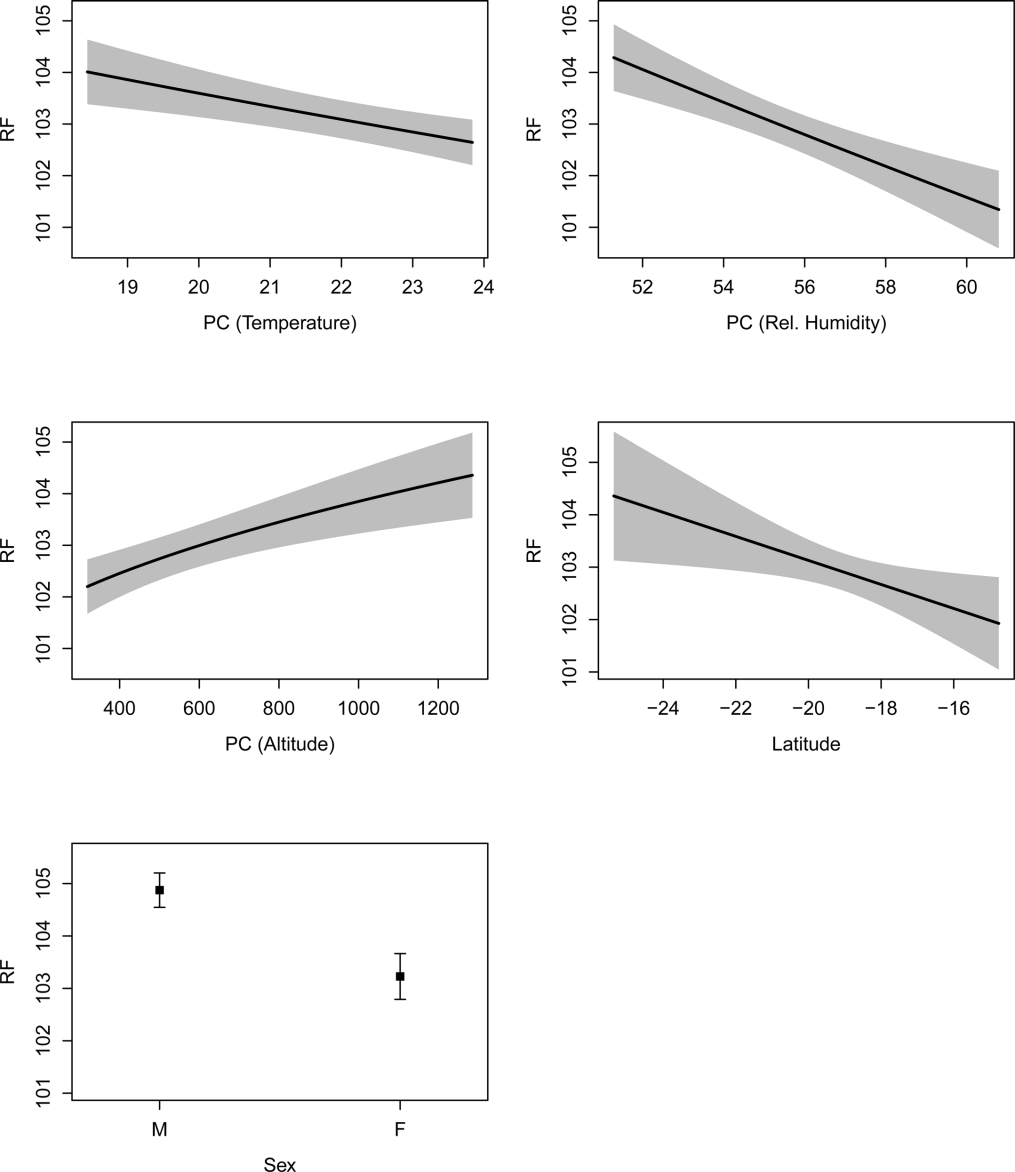
Predictive modelling plots showing how resting frequency (RF) in *Rhinolophus swinnyi* responded to environmental variation and sex. Abbreviations: e.g., PC (Altitude) represents a principle component generated by fixing mean-annual-temperature and relative humidity (rel.humidity) to their across-site-means whilst altitude is allowed to vary. The shaded areas and error bars represent 95% confidence limits. The final best model (with only variables shown significant after ANOVA on the best model) was used for the modelling.

The effects of each of the climatic variables (RH, AnnTemp and Alt) were isolated by holding (controlling) the others constant at the across-site mean. The climatic variables exhibited predominantly linear relationships with RF across the different habitats, with the exceptions of non-linear relationships in AnnTemp and Alt for *R*. *simulator* (Figs 3 and 4). This uncoupled effect appeared to be generally stronger in *R*. *swinnyi* than in *R*. *simulator* (Figs 3 and 4).These predictive modelling results may also indicate that each climatic variable was associated with variation in RF in the context of the other two. Importantly, our results could not attribute divergence in RF to climatic variables alone, as gender and region (North–South spatial structuring; Lat) were also associated with variation in RF (Table 1; Figs 3 and 4). RF increased significantly with a southward increase in distance for both species (Figs 3 and 4); whereas longitude did not explain significant variation in RF (Table 1).

The Mantel test showed variation in RF was positively associated with geographic distances in *R*. *simulator*: differences in resting frequency were larger among pairs of sampled sites that were further from each other than those that were nearer (Monte-Carlo test Observation: 0.647, 10 000 replicates; *P* = 0.002). The association between RF differences and geographic distances was not significant for *R*. *swinnyi* (MC test Observation: 0.550; 10 000 replicates, *P* = 0.059).

Sexual dimorphism was evident in both species but stronger in *R*. *swinnyi* than *R*. *simulator* (overall across all populations; Figs 3 and 4). At most sites with both sexes, males used higher RFs although these were not always statistically significant for both species. Females never used higher frequencies than males except at MT in *R*. *simulator* and at MC in *R*. *swinnyi* (Figs 3 and 4; S1 Table 1a and b). *R*. *simulator* males used higher RFs than females at three sites i.e. at CC, GKC & MC and in *R*. *swinnyi* males used higher RFs at two sites i.e., at KP and OD (S1 Table 1a and b).

## Discussion

Body size (FA) was not correlated with RF in either *R*. *swinnyi* or *R*. *simulator* suggesting RF variation was not the result of climate-mediated variation in body size as proposed by James’ Rule. In accordance with the Sensory Drive Hypothesis, RF in *R*. *swinnyi* was predominantly climate driven. However, this was not the case with *R*. *simulator*. Instead, RF in this species was predominantly latitude driven. Both species showed significant spatial structuring by latitude, and sexual dimorphism (which was stronger in *R*. *swinnyi*). The effect of latitude suggests that other climatic and environmental variables not considered here may also exert an influence.

The extent to which each species responded to climatic variables was dependent on its specific echolocation frequency. These observed changes were larger in *R*. *swinnyi* than *R*. *simulator* even though both species are exposed to the same climate (where they co-occurred) or to similar RH and AnnTemp (47–61% RH; 17.7–21.5°C AnnTemp and 51–61% RH; 18.5–23.8°C AnnTemp for *R*. *simulator* and *R*. *swinnyi*, respectively; S1 Table 1a & b). This was probably because atmospheric attenuation is more pronounced at the higher call frequencies used by *R*. *swinnyi*. Thus, lowering call frequency in response to higher RH is probably crucial for *R*. *swinnyi* to maintain comparable detection distances (mean = 6.13 ± 0.25 m; range = 5.7–6.5 m) across its distributional range.

The reason for the influence of latitude on RF (besides its influence exerted through AnnTemp, RH and Alt) in both species is difficult to ascertain at this stage. A possible explanation for the latitudinal cline in RF could be the effect of isolation by distance and vicariance because the effect of distance between populations was stronger for *R*. *simulator*, before controlling for spatial autocorrelation (S3 Fig). Surprisingly, very few studies [20] have found such correlations between geographic co-ordinates and RF; reviewed in Jiang *et al*. [49].

The strong north-to-south structuring in the RFs therefore raised a possibility of the existence of a latent variable in latitude, besides the tested variables in the PCs (RH, Alt and AnnTemp). Atmospheric pressure, for example, was found to be a significant influence on call frequency in Gillam [35]. Unfortunately, we could not get reliable data to directly test the influence of atmospheric pressure (mean annual averages over 20–50 years) on RF comparable to our other climatic variables (RH and AnnTemp).

Our results on the influence of climatic factors on geographic variation in call frequency generally support those of other studies but with some notable differences. Studies on *Hipposideros ruber* [19] and *Rhinolophus pusillus* [50] in the tropics of Africa and Asia, respectively, found support for an association between mean annual rainfall and RF, where mean annual rainfall was used as a proxy for RH. However, Odendaal *et al*. [32] found no association between RH and RF in *R*. *capensis*, although the species occurred in biomes ranging from desert to forest but across which, surprisingly, RH did not vary significantly. However, there appeared to be a correlation between RF and mean annual precipitation (DSJ, personal observation). These discrepancies between studies and between species within studies (reported here) suggest that the atmospheric attenuation experienced by bats is the result of a complex interaction between local temperature, humidity, atmospheric pressure and rainfall, as well as the frequency of the acoustic signal. All of these should be considered in attempts to understand how climatic factors drive acoustic variation.

The small range in RFs across populations of *R*. *swinnyi* (4 kHz) and *R*. *simulator* (7 kHz) raises the possibility that variation may not be ecologically relevant. The maximum difference in detection range across the frequencies used by *R*. *swinnyi* and *R*. *simulator* was 1.0 m and 0.8 m, respectively. Differences in detection ranges of 1m are likely to make considerable differences in the detection of prey and the avoidance of obstacles at increased flight speeds. Studies on exactly how these bats vary echolocation and flight speeds are sorely needed. The advent of multiple microphone arrays will facilitate this.

It is possible that part of the variation in RF could be driven by the presence of other horseshoe bat species in multi-species assemblages e.g., partitioning of frequency bands so that each species in bat assemblages has its own private band allowing more effective intraspecific communication (Acoustic Communication Hypothesis) [22,51,52]. If so, it might explain the absence of a correlation between body size and RF. At Lobatse (Botswana) where *R*. *simulator* is syntopic (occurring in the same cave) with only one other species of rhinolophid, *R*. *clivosus* (92 kHz; DSJ unpublished data) it calls at 85 kHz (S1 Table 1a). However, where *R*. *simulator* is syntopic with several species of rhinolophids including *R*. *blasii* which echolocates at 86 kHz [24] it calls at 80 kHz (S1 Table 1a) which ensures that its call frequency does not overlap with that of *R*. *blasii*. Confirmation of this would require more detailed analyses comparing echolocation call frequencies of both *R*. *simulator* and *R*. *swinnyi* and several other rhinolophid species with overlapping geographic distributions in situations of syntopy and allopatry. It is also note-worthy that variations in RF may be driven by sexual selection in which female choice and male-male competition [53,54] may drive the divergence in RF [6,49].

Males generally called at higher frequencies than females, although the differences were not always significant (S1 Table 1a and b). Our current data do not allow us to test potential explanations for sexual dimorphism or why it varies across localities. Future analyses could focus on call parameters other than frequency (e.g., slope and minimum frequency of the frequency modulated component of the call, duration and inter-pulse interval) to provide more detail on sexual differences in calls. This should provide further insight into the potential communicative function of echolocation and how environmental/climatic conditions may influence information exchange between different sexes and how such processes may contribute to geographic variation.

The isolation by distance patterns we obtained from the Mantel test indicated that there is differential gene flow between populations, at least in *R*. *simulator*, and that environmental factors may not be solely responsible for the variation in RF amongst populations. If populations are sufficiently small then stochastic factors such as genetic drift in combination with reduced gene flow may exert an influence [55]. However, average geographic distances were in fact similar for both species but slightly lower for *R*. *simulator* than for *R*. *swinnyi* (mean mahalanobis distance of coordinates = 5.09 and 5.26, respectively) and suggest that other barriers besides distance may reduce gene flow between populations, at least in *R*. *simulator*.

The contribution of atmospheric conditions to variation in acoustic signals may not be restricted to animals using high frequency acoustic signals. There is also some support for atmospheric attenuation contributing to geographic variation in low frequency bird song [18] suggesting that lineage diversification may be driven by habitat-mediated differences in communication signals in a variety of terrestrial (and perhaps also marine) taxa that rely on acoustic signals for orientation, food and mate acquisition. Sensory drive may have a greater effect on the generation of biodiversity than is currently appreciated. However, evidence that habitat driven variation in acoustic signals lead directly to lineage diversification is sorely needed. Climate driven changes in acoustic signals, as shown in our study, may have implications for the understanding not only of lineage diversification, but also of how organisms may respond to climate change over space and time.

## Supporting Information

S1 Fig Typical echolocation calls for a) *Rhinolophus swinnyi* and b) *Rhinolophus simulator*. (TIF)

S2 Fig Residual distribution and model validation graphs for *Rhinolophus simulator* (top panel) and *Rhinolophus swinnyi* (bottom panel). Within each panel; from the top we show the linear-mixed-effects model as a stand-alone; below this we show the best model, i.e., after all spatial autocorrelation structures with and without study sites as a random effect have been tested. In this case, both species showing the best model structure as linear mixed effects with study sites as random effects.
(TIF)

S3 Fig Spline correlograms of the residuals (with 95% confidence intervals) from a linear mixed effects model with study sites as random effects, including all predictor variables. *Rhinolophus simulator* and *Rhinolophus swinnyi* (bottom). The correlation is measured in Moran’s I spatial auto-correlation index [42].
(TIF)

S1 Table Means and standard deviations for phenotypic and environmental variables. (DOCX)

## Abbreviations

RF: Resting Frequency of the echolocation call signal
FA: Forearm length
RH: Relative humidity
AnnTemp: Mean Annual Temperature
Alt: Altitude

## References

[1] Magurran AE (1998) Population differentiation without speciation. Philosophical Transactions of the Royal Society of London Series B: Biological Sciences 353: 275–286.

[2] Lomolino MV, Sax DF, Riddle BR, Brown JH (2006) The island rule and a research agenda for studying ecogeographical patterns. Journal of Biogeography: Wiley-Blackwell. pp. 1503–1510.

[3] Morrone JJ (2009) Evolutionary biology: an intergrative approach with case studies Columbia: Columbia University Press.

[4] Barton NH, Charlesworth B (1984) Genetic revolutions, founder effects, and speciation. Annual Review of Ecology and Systematics: 133–164.

[5] Gübitz T, Thorpe RS, Malhotra A (2005) The dynamics of genetic and morphological variation on volcanic islands. Proceedings of the Royal Society of London B: Biological Sciences 272: 751–757. 10.1098/rspb.2004.3018 PMC1602046 15870037

[6] Puechmaille SJ, Borissov IM, Zsebok S, Allegrini B, Hizem M, Kuenzel S, et al (2014) Female mate choice can drive the evolution of high frequency echolocation in bats: a case study with *Rhinolophus mehelyi*. PLoS ONE 9: e103452 10.1371/journal.pone.0103452 25075972 PMC4116191

[7] Bastian A, Jacobs DS (2015) Listening carefully: increased perceptual acuity for species discrimination in multispecies signalling assemblages. Animal Behaviour 101: 141–154.

[8] Luo J, Koselj K, Zsebok S, Siemers BM, Goerlitz HR (2013) Global warming alters sound transmission: differential impact on the prey detection ability of echolocating bats. Journal Of The Royal Society, Interface / The Royal Society 11: 20130961–20130961. 10.1098/rsif.2013.0961 24335559 PMC3869170

[9] Wilczynski W, Ryan MJ (1999) Geographic variation in animal communication systems. Geographic Diversification of Behavior: An Evolutionary Perspective: 234–261.

[10] Endler JA (1992) Signals, signal conditions, and the direction of evolution. American Naturalist: S125–S153.

[11] Ey E, Fischer J (2009) The “acoustic adaptation hypothesis”—a review of the evidence from birds, anurans and mammals. Bioacoustics 19: 21–48.

[12] Barclay RM (1982) Interindividual use of echolocation calls: eavesdropping by bats. Behavioral Ecology and Sociobiology 10: 271–275.

[13] Fenton MB, Audet D, Obrist MK, Rydell J (1995) Signal strength, timing, and self-deafening; the evolution of echolocation in bats. Paleobiology 21: 229–242.

[14] Knörnschild M, Jung K, Nagy M, Metz M, Kalko E (2012) Bat echolocation calls facilitate social communication. Proceedings of the Royal Society B: Biological Sciences 279: 4827–4835. 10.1098/rspb.2012.1995 23034703 PMC3497101

[15] Fenton MB (2003) Eavesdropping on the echolocation and social calls of bats. Mammal Review 33: 193.

[16] Kazial KA, Masters WM (2004) Female big brown bats, *Eptesicus fuscus*, recognize sex from a caller's echolocation signals. Animal Behaviour 67: 855–863.

[17] Lawrence BD, Simmons JA (1982) Measurements of atmospheric attenuation at ultrasonic frequencies and the significance for echolocation by bats. The Journal Of The Acoustical Society Of America 71: 585–590. 7085967 10.1121/1.387529

[18] Snell-Rood EC (2012) The effect of climate on acoustic signals: does atmospheric sound absorption matter for bird song and bat echolocation? Journal of the Acoustical Society of America 131: 1650–1658. 10.1121/1.3672695 22352535

[19] Guillén, Juste B, Ibáñez (2000) Variation in the frequency of the echolocation calls of *Hipposideros ruber* in the Gulf of Guinea: an exploration of the adaptive meaning of the constant frequency value in rhinolophoid CF bats. Journal of Evolutionary Biology 13: 70–80.

[20] Jiang T, Liu R, Metzner W, You Y, Li S, Liu S, et al (2010) Geographical and individual variation in echolocation calls of the intermediate leaf-nosed bat, Hipposideros larvatus. Ethology 116: 691–703.

[21] Jones G (1999) Scaling of echolocation call parameters in bats. Journal of Experimental Biology 202: 3359–3367. 10562518 10.1242/jeb.202.23.3359

[22] Jacobs DS, Barclay RMR, Walker MH (2007) The allometry of echolocation call frequencies of insectivorous bats: why do some species deviate from the pattern? Oecologia 152: 583–594. 17345101 10.1007/s00442-007-0679-1

[23] James FC (1970) Geographic size variation in birds and Its relationship to climate. Ecology 51: 365–390.

[24] Schoeman MC, Jacobs DS (2008) The relative influence of competition and prey defenses on the phenotypic structure of insectivorous bat ensembles in southern Africa. PLoS ONE 3: 1–10. 10.1371/journal.pone.0003715 PMC2579324 19005563

[25] Monadjem A, Taylor PJ, Cotterill FPD, Schoeman CM (2010) Bats of southern and central Africa: a biogeographic and taxonomic synthesis Johannesburg: Wits University Press.

[26] Olson DM, Dinerstein E, Wikramanayake ED, Burgess ND, Powell GV, Underwood EC, et al (2001) Terrestrial Ecoregions of the World: A New Map of Life on Earth: A new global map of terrestrial ecoregions provides an innovative tool for conserving biodiversity. BioScience 51: 933–938.

[27] Gannon WL, Sikes RS (2007) Guidelines of the American Society of Mammalogists for the use of wild mammals in research. Journal Of Mammalogy 88: 809–823. 10.1093/jmammal/gyw078 PMC5909806 29692469

[28] Aegerter J, Heritage SN (2005) The extent and frequency of European bat Lyssavirus (EBLV2) in Scotland as determined by surveillance testing of sero-prevalence and tissue sampling: Scottish Natural Heritage.

[29] Kunz TH, Parsons S (2009) Ecological and behavioral methods for the study of bats: Johns Hopkins University Press.

[30] Racey PA (1974) Ageing and assessment of reproductive status of Pipistrelle bats, *Pipistrellus pipistrellus*. Journal of Zoology 173: 264–271. 4468893 10.1111/j.1469-7998.1974.tb03136.x

[31] Anthony ELP (1988) Age determination in bats In: Kunz TH, editor. Ecological and behavioral methods for the study of bats. Washington DC: Smithsonian Institution Press pp. 47–58.

[32] Odendaal LJ, Jacobs DS, Bishop JM (2014) Sensory trait variation in an echolocating bat suggests roles for both selection and plasticity. BMC Evolutionary Biology 14: 60–60. 10.1186/1471-2148-14-60 24674227 PMC3986686

[33] Siemers BM, Beedholm K, Dietz C, Dietz I, Ivanova T (2005) Is species identity, sex, age or individual quality conveyed by echolocation call frequency in European horseshoe bats? Acta Chiropterologica 7: 259–274.

[34] Schnitzler HU (1987) Echoes of fluttering insects: information from echolocating bats In: Fenton MB, Racey PA, Rayner JMV, editors. Recent advances in the study of bats. Cambridge: Cambridge University Press.

[35] Gillam E, McCracken G, Westbrook J, Lee Y-F, Jensen M, Balsley BB (2009) Bats aloft: variability in echolocation call structure at high altitudes. Behavioral Ecology and Sociobiology 64: 69–79.

[36] Zuckerwar AJ (2002) Handbook of the Speed of Sound in Real Gases: Elsevier Science & Technology Books.

[37] Stilz WP, Schnitzler HU (2012) Estimation of the acoustic range of bat echolocation for extended targets. The Journal of the Acoustical Society of America 132: 1765–1775. 10.1121/1.4733537 22978903

[38] Schuchmann M, Siemers BM (2010) Variability in echolocation call intensity in a community of Horseshoe Bats: a role for resource partitioning or communication? PLoS ONE 5: e12842 10.1371/journal.pone.0012842 20862252 PMC2941460

[39] Sternheim MM, Kane JW (1986) General physics New York: Wiley.

[40] Dormann CF, Elith J, Bacher S, Buchmann C, Carl G, Carré G, et al (2013) Collinearity: a review of methods to deal with it and a simulation study evaluating their performance. Ecography 36: 27–46.

[41] BjØrnstad ON, Falck W (2001) Nonparametric spatial covariance functions: estimation and testing. Environmental and Ecological Statistics 8: 53–70.

[42] Cliff AD, Ord K (1970) Spatial autocorrelation: a review of existing and new measures with applications. Economic Geography: 269–292.

[43] Tobler WR (1970) A computer movie simulating urban growth in the Detroit region. Economic Geography: 234–240.

[44] Zuur A, Ieno EN, Walker N, Saveliev AA, Smith GM (2009) Mixed effects models and extensions in ecology with R: Springer Science & Business Media.

[45] Dray S, Dufour AB (2007) The ade4 package: implementing the duality diagram for ecologists. Journal of Statistical Software 22: 1–20.

[46] Barnard GA (1963) Discussion of Professor Bartlett’s paper. JR Statist Soc B 25: 294.

[47] Kormann U, Rösch V, Batáry P, Tscharntke T, Orci KM, Samu F, et al (2015) Local and landscape management drive trait‐mediated biodiversity of nine taxa on small grassland fragments. Diversity and Distributions: 1–14.

[48] Crawley MJ (2012) The R book: John Wiley & Sons.

[49] Jiang T, Wu H, Feng J (2015) Patterns and causes of geographic variation in bat echolocation pulses. Integrative Zoology 10: 241–256. 10.1111/1749-4877.12129 25664901

[50] Jiang T, Metzner W, You Y, Liu S, Lu G, Li S, et al (2010) Variation in the resting frequency of *Rhinolophus pusillus* in Mainland China: effect of climate and implications for conservation. The Journal Of The Acoustical Society Of America 128: 2204–2211. 10.1121/1.3478855 20968390 PMC2981126

[51] Duellman WE, Pyles RA (1983) Acoustic resource partitioning in anuran communities. Copeia: 639–649.

[52] Heller K-G, Helversen O (1989) Resource partitioning of sonar frequency bands in rhinolophoid bats. Oecologia 80: 178–186. 28313104 10.1007/BF00380148

[53] Andersson MB (1994) Sexual selection: Princeton University Press.

[54] Podos J, Warren PS (2007) The evolution of geographic variation in birdsong. Advances in the Study of Behavior 37: 403–458.

[55] Hutchison DW, Templeton AR (1999) Correlation of pairwise genetic and geographic distance measures: inferring the relative influences of gene flow and drift on the distribution of genetic variability. Evolution: 1898–1914. 28565459 10.1111/j.1558-5646.1999.tb04571.x

