# Supplementary figures and images for: Sensory Drive Mediated by Climatic Gradients Partially Explains Divergence in Acoustic Signals in Two Horseshoe Bat Species, Rhinolophus swinnyi and Rhinolophus simulator
Source: PLoS One. 2016 Jan 27;11(1):e0148053. doi: 10.1371/journal.pone.0148053 (PMC4729529; doi:10.1371/journal.pone.0148053)

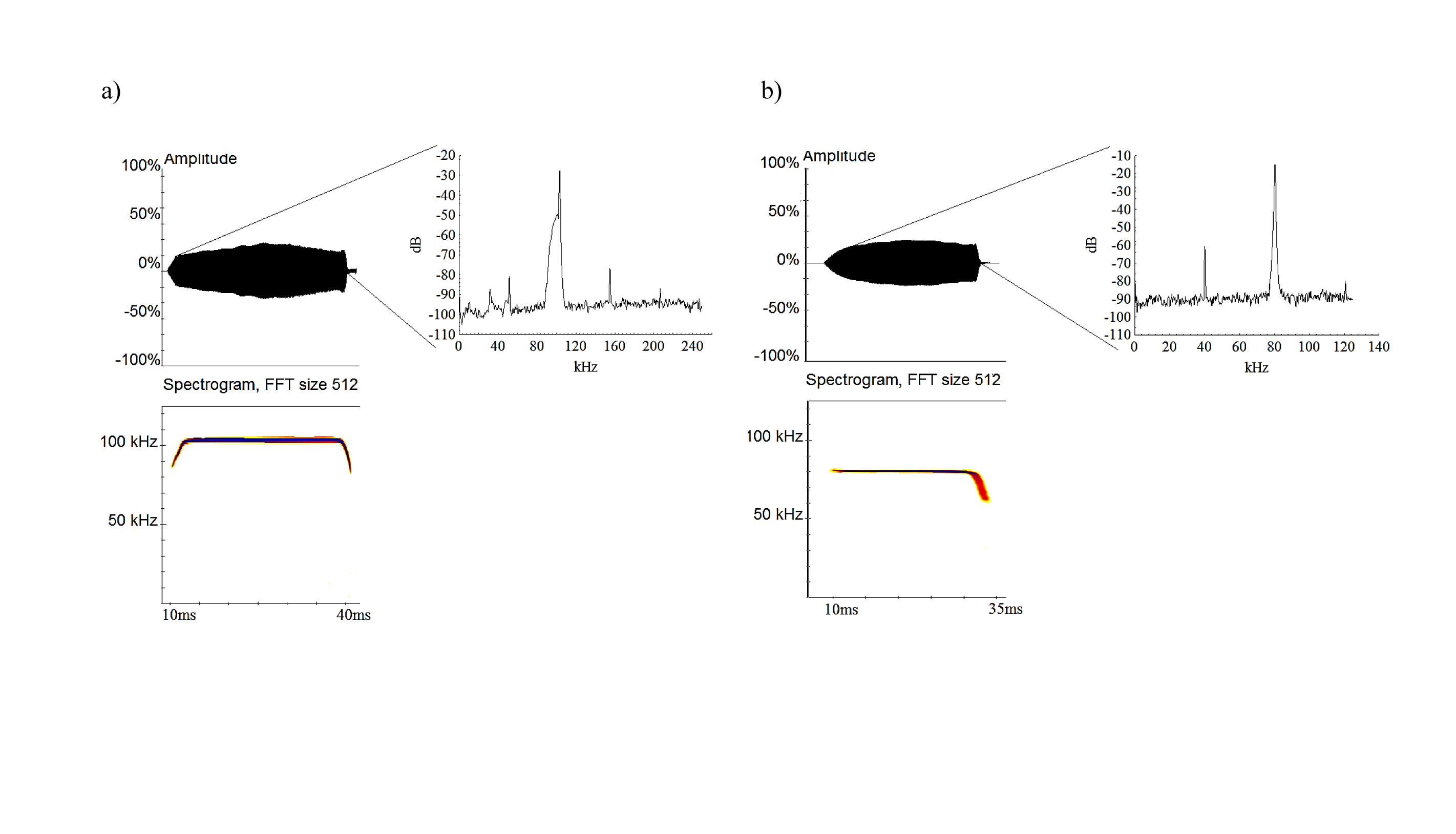

Supplement: S1 Fig — (TIF) [file pone.0148053.s001.tif]

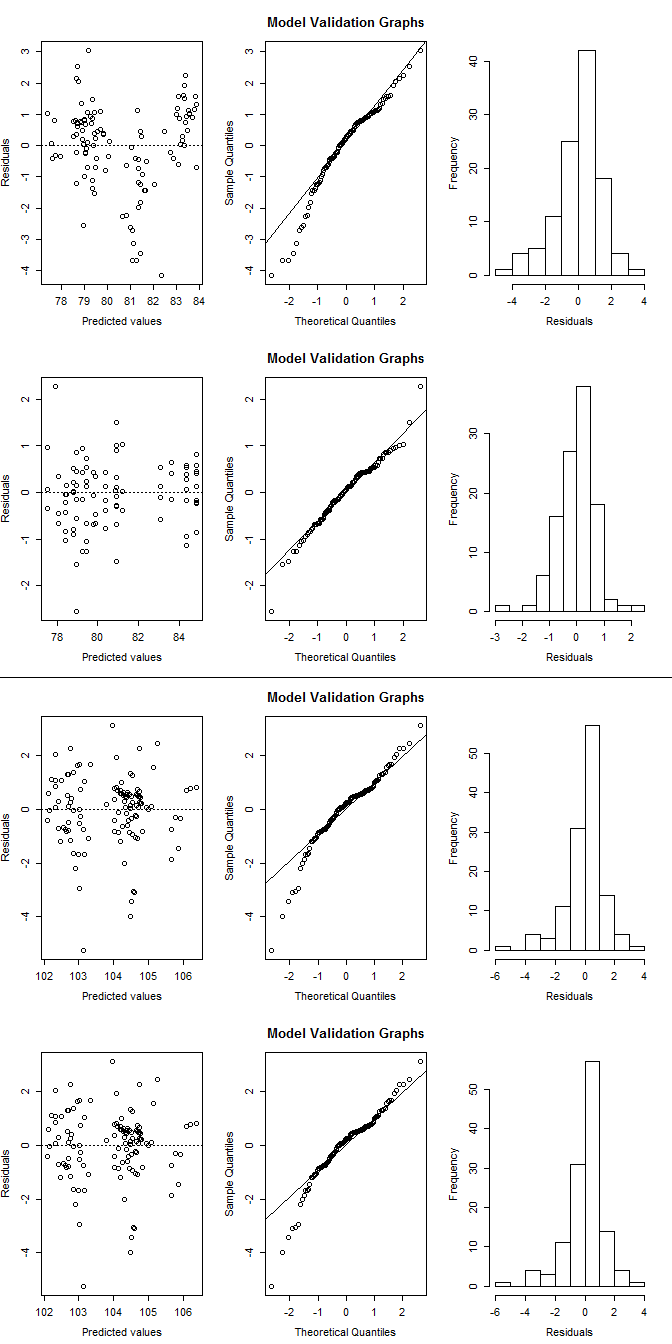

Supplement: S2 Fig — Within each panel; from the top we show the linear-mixed-effects model as a stand-alone; below this we show the best model, i.e., after all spatial autocorrelation structures with and without study sites as a random effect have been tested. In this case, both species showing the best model structure as linear mixed effects with study sites as random effects. (TIF) [file pone.0148053.s002.tif]

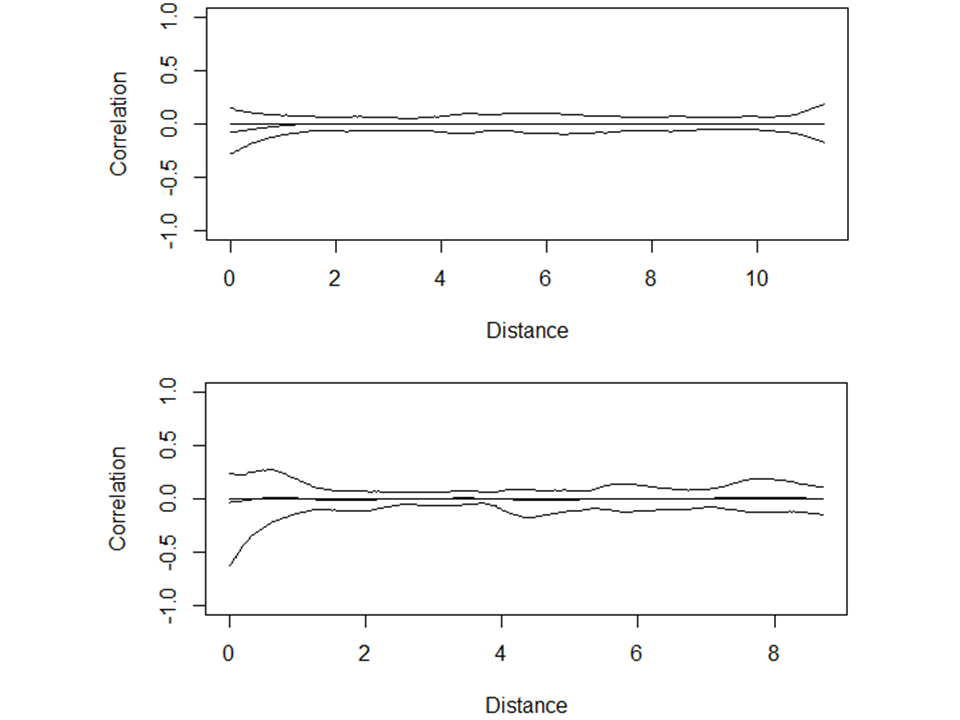

Supplement: S3 Fig — Rhinolophus simulator and Rhinolophus swinnyi (bottom). The correlation is measured in Moran’s I spatial auto-correlation index [42]. (TIF) [file pone.0148053.s003.tif]
