# Supplementary material for: Sensory Drive Mediated by Climatic Gradients Partially Explains Divergence in Acoustic Signals in Two Horseshoe Bat Species, Rhinolophus swinnyi and Rhinolophus simulator
Source: PLoS One. 2016 Jan 27;11(1):e0148053. doi: 10.1371/journal.pone.0148053 (PMC4729529; doi:10.1371/journal.pone.0148053)
Supplement: S1 Table — (DOCX) [file pone.0148053.s004.docx]

**Table 1 a.** Phenotypic parameters and environmental variables (Mean ± SD) for *R. simulator* from different sites within southern Africa.

| Site/Sex | N | FA | ±SD | RF | ±SD | Dur(ms) | ±SD | Lat | Long | Alt(m) | RH(%) | AnnTemp |
| --- | --- | --- | --- | --- | --- | --- | --- | --- | --- | --- | --- | --- |
| CC | **8** | **44.29** | **1.07** | **80.14** | **0.59** | **18.07** | **3.44** | **17.36** | **240.17** | **1171.23** | **53.23** | **19.12** |
| F | 4 | 44.83 | 1.12 | 79.75 | 0.53 | 16.13 | 2.02 | 17.36 | 119.83 | 1145.02 | 52.70 | 19.10 |
| M | 4 | 43.75 | 0.79 | 80.53 | 0.38 | 20.00 | 3.68 | 17.36 | 120.34 | 1197.44 | 53.77 | 19.14 |
| DM | **22** | **44.45** | **1.21** | **79.18** | **0.95** | **14.94** | **1.86** | **16.83** | **687.14** | **1171.55** | **54.19** | **18.54** |
| F | 12 | 44.92 | 1.15 | 78.93 | 1.05 | 15.35 | 1.49 | 16.83 | 374.81 | 1171.55 | 54.19 | 18.54 |
| M | 10 | 43.88 | 1.07 | 79.49 | 0.76 | 14.45 | 2.21 | 16.83 | 312.34 | 1171.55 | 54.19 | 18.54 |
| GKC | **10** | **45.50** | **1.16** | **83.29** | **0.67** |  |  | **24.63** | **276.69** | **1180.46** | **47.52** | **18.84** |
| F | 6 | 45.75 | 1.43 | 82.88 | 0.46 |  |  | 24.63 | 166.01 | 1180.46 | 47.52 | 18.84 |
| M | 4 | 45.13 | 0.56 | 83.92 | 0.34 |  |  | 24.63 | 110.67 | 1180.46 | 47.52 | 18.84 |
| KL | **6** | **45.12** | **0.97** | **77.78** | **0.54** | **24.82** | **5.32** | **14.74** | **161.43** | **1285.77** | **57.94** | 20.04 |
| F | 3 | 44.53 | 0.83 | 77.77 | 0.67 | 22.78 | 5.51 | 14.74 | 80.72 | 1285.77 | 57.94 | 20.04 |
| M | 3 | 45.70 | 0.80 | 77.80 | 0.53 | 27.88 | 4.62 | 14.74 | 80.72 | 1285.77 | 57.94 | 20.04 |
| LOB | **18** | **45.03** | **1.04** | **84.61** | **0.64** | **22.20** | **3.89** | **25.24** | **461.91** | **1264.77** | **46.49** | **18.24** |
| F | 8 | 45.48 | 1.09 | 84.26 | 0.66 | 21.98 | 2.70 | 25.24 | 205.29 | 1264.77 | 46.49 | 18.24 |
| M | 10 | 44.68 | 0.90 | 84.90 | 0.50 | 22.38 | 4.77 | 25.24 | 256.61 | 1264.77 | 46.49 | 18.24 |
| MC | **8** | **45.28** | **1.27** | **79.53** | **0.77** | **15.04** | **2.50** | **17.90** | **234.93** | **877.84** | **51.29** | **21.55** |
| F | 4 | 45.73 | 1.27 | 79.25 | 0.95 | 15.73 | 3.20 | 17.90 | 117.47 | 877.84 | 51.29 | 21.55 |
| M | 4 | 44.83 | 1.26 | 79.80 | 0.52 | 14.35 | 1.75 | 17.90 | 117.47 | 877.84 | 51.29 | 21.55 |
| MM | **4** | **44.00** | **1.15** | **81.25** | **0.66** | **31.42** | **4.33** | **18.88** | **130.90** | **1215.77** | **60.84** | **17.68** |
| M | 4 | 44.00 | 1.15 | 81.25 | 0.66 | 31.42 | 4.33 | 18.88 | 130.90 | 1215.77 | 60.84 | 17.68 |
| MT | **10** | **45.84** | **2.25** | **78.32** | **0.76** | **28.04** | **7.19** | **20.55** | **285.08** | **1313.63** | **48.74** | **18.24** |
| F | 1 | 47.30 |  | 80.20 |  | 31.65 |  | 20.55 | 28.51 | 1313.63 | 48.74 | 18.24 |
| M | 9 | 45.68 | 2.33 | 78.11 | 0.40 | 27.64 | 7.51 | 20.55 | 256.57 | 1313.63 | 48.74 | 18.24 |
| SH | **9** | **44.14** | **1.49** | **78.79** | **0.51** | **31.00** | **13.08** | **15.64** | **254.59** | **1116.28** | **53.64** | **20.98** |
| M | 9 | 44.14 | 1.49 | 78.79 | 0.51 | 31.00 | 13.08 | 15.64 | 254.59 | 1116.28 | 53.64 | 20.98 |
| SUD | **16** | **44.47** | **1.31** | **80.74** | **0.81** | **23.83** | **8.85** | **25.28** | **489.58** | **1093.19** | **58.79** | **17.74** |
| F | 5 | 44.08 | 2.05 | 80.34 | 0.53 | 27.05 | 8.29 | 25.12 | 151.95 | 1155.89 | 58.12 | 17.51 |
| M | 11 | 44.65 | 0.89 | 80.92 | 0.87 | 22.36 | 9.07 | 25.36 | 337.63 | 1064.69 | 59.10 | 17.85 |
| Total N | 111 |  |  |  |  |  |  |  |  |  |  |  |

Abbreviations: AnnPrec = Mean Annual Precipitation (mm), AnnTemp = Mean Annual Temperature (°C), RH = Relative Humidity (%), Lat = Latitude, Long = Longitude, N = sample size, M = Males, F = Females, SD = standard deviation, RF = resting frequency of the echolocation call (kHz), Dur = Call Duration (ms), FA = Forearm length (cm), Mass = Mass (g). Locality codes, CC = Chinhoyi Cave, DM = Dambanzara, Zimbabwe; GKC = Gatkop Cave, South Africa, KL = Kalenda, Zambia; LOB = Lobatse Estate, Botswana; MC = Mabura Cave, Zimbabwe; MM = Monaci Mine, Zimbabwe; MT = Matobo, Zimbabwe; SH = Shimabala, Zambia; SUD = Sudwala Cave, South Africa.

**Table 1 b.** Phenotypic parameters and environmental variables (Mean ± SD) for *R. swinnyi* from different sites within southern Africa.

| Site/Sex | N | FA | ±SD | RF | ±SD | Dur(ms) | ±SD | Lat | Long | Alt(m) | RH(%) | AnnTemp |
| --- | --- | --- | --- | --- | --- | --- | --- | --- | --- | --- | --- | --- |
| CC | **4** | **41.43** | **1.34** | **106.55** | **0.91** | **24.94** | **11.08** | **17.36** | **120.00** | **1162.49** | **53.06** | **19.11** |
| F | 1 | 43.20 |  | 105.20 |  | 18.12 |  | 17.36 | 29.96 | 1145.02 | 52.70 | 19.10 |
| M | 3 | 40.83 | 0.76 | 107.00 | 0.20 | 27.21 | 12.38 | 17.36 | 90.04 | 1168.31 | 53.17 | 19.12 |
| DM | **4** | **42.13** | **0.76** | **104.23** | **1.99** | **17.55** | **2.35** | **16.83** | **124.94** | **1171.55** | **54.19** | **18.54** |
| F | 3 | 42.47 | 0.40 | 103.77 | 2.16 | 17.45 | 2.86 | 16.83 | 93.70 | 1171.55 | 54.19 | 18.54 |
| M | 1 | 41.10 |  | 105.60 |  | 17.85 |  | 16.83 | 31.23 | 1171.55 | 54.19 | 18.54 |
| JET | **21** | **42.17** | **1.81** | **103.97** | **1.98** | **30.48** | **4.23** | **20.94** | **658.97** | **446.20** | **54.12** | **22.25** |
| F | 7 | 42.44 | 1.31 | 102.52 | 1.51 | 31.53 | 5.71 | 20.94 | 219.66 | 446.20 | 54.12 | 22.25 |
| M | 14 | 42.04 | 2.04 | 104.59 | 1.86 | 30.03 | 3.59 | 20.94 | 439.31 | 446.20 | 54.12 | 22.25 |
| KL | **8** | **42.91** | **1.18** | **102.83** | **0.77** | **29.34** | **6.85** | **14.85** | **216.57** | **1264.54** | **57.40** | **20.16** |
| F | 7 | 42.96 | 1.27 | 102.66 | 0.65 | 30.03 | 7.09 | 14.87 | 189.66 | 1261.51 | 57.33 | 20.18 |
| M | 1 | 42.60 |  | 104.00 |  | 24.47 |  | 14.74 | 26.91 | 1285.77 | 57.94 | 20.04 |
| KP | **14** | **45.49** | **1.36** | **103.28** | **1.55** | **44.02** | **16.01** | **16.40** | **432.00** | **683.88** | **53.20** | **21.60** |
| F | 9 | 45.61 | 1.29 | 102.63 | 1.51 | 47.06 | 18.90 | 16.40 | 277.72 | 683.88 | 53.20 | 21.60 |
| M | 5 | 45.26 | 1.61 | 104.44 | 0.78 | 38.55 | 7.75 | 16.40 | 154.29 | 683.88 | 53.20 | 21.60 |
| MC | **9** | **41.70** | **0.50** | **104.98** | **1.14** | **18.91** | **3.35** | **17.90** | **264.30** | **877.84** | **51.29** | **21.55** |
| F | 5 | 41.78 | 0.46 | 105.24 | 1.42 | 17.83 | 4.24 | 17.90 | 146.83 | 877.84 | 51.29 | 21.55 |
| M | 4 | 41.60 | 0.60 | 104.65 | 0.72 | 20.26 | 1.28 | 17.90 | 117.47 | 877.84 | 51.29 | 21.55 |
| OD | **33** | **41.25** | **0.97** | **103.50** | **1.26** | **22.33** | **12.09** | **18.95** | **1065.96** | **1057.54** | **60.79** | **18.54** |
| F | 14 | 41.14 | 1.07 | 102.56 | 0.99 | 23.63 | 13.96 | 18.94 | 452.87 | 1056.40 | 60.81 | 18.55 |
| M | 19 | 41.34 | 0.90 | 104.19 | 0.97 | 21.38 | 10.80 | 18.95 | 613.09 | 1058.38 | 60.78 | 18.54 |
| PA | **33** | **40.94** | **0.74** | **104.31** | **1.52** | **27.37** | **7.77** | **22.42** | **1024.18** | **319.03** | **51.76** | **23.85** |
| F | 7 | 41.03 | 0.96 | 102.59 | 2.25 | 29.81 | 10.04 | 22.42 | 217.25 | 319.03 | 51.76 | 23.85 |
| M | 26 | 40.92 | 0.69 | 104.78 | 0.82 | 26.71 | 7.14 | 22.42 | 806.93 | 319.03 | 51.76 | 23.85 |
| Total N | 126 |  |  |  |  |  |  |  |  |  |  |  |

Abbreviations: JET = Jiri Estate – Triangle, Zimbabwe; KP = Kapatamukombe, Zimbabwe; OD = Odzi German Shafts, Zimbabwe; PA = Pafuri, South Africa. All other abbreviations are given in table 1 b.
